# Pressing of Functionalized Polymer Composite Materials to Improve Mössbauer Measurement Signals

**DOI:** 10.3390/polym16101311

**Published:** 2024-05-07

**Authors:** Justus Pawlak, Jules Brehme, Maximilian Seydi Kilic, Kevin Tran, Justin Koch, Mohammad Beyki, Ralf Sindelar, Robert Patzke, Franz Renz

**Affiliations:** 1 Institute of Inorganic Chemistry, Leibniz Universität Hannover, Callinstraße 7, 30167 Hannover, Germany; justus.pawlak@acd.uni-hannover.de (J.P.); or jules.brehme@hs-hannover.de (J.B.); maximilian.kilic@acd.uni-hannover.de (M.S.K.); kevin.tran@acd.uni-hannover.de (K.T.); justin.koch@stud.uni-hannover.de (J.K.); 2 Hannover School for Nanotechnology, Laboratorium für Nano-und Quantenengineering (LNQE), Leibniz Universität Hannover, Schneiderberg 39, 30167 Hannover, Germany; mohammad.beyki@hs-hannover.de (M.B.); ralf.sindelar@hs-hannover.de (R.S.); robert.patzke@hs-hannover.de (R.P.); 3 Faculty I, Hochschule Hannover, University of Applied Science an Arts, Ricklinger Stadtweg 120, 30459 Hannover, Germany; 4 Faculty II, Hochschule Hannover, University of Applied Science an Arts, Ricklinger Stadtweg 120, 30459 Hannover, Germany

**Keywords:** nanofiber, electrospinning, Mössbauer, molecular switch, triazole complexes, spin crossover, pellet, composites, coordination chemistry

## Abstract

Coordination compounds, like iron(II) triazole complexes, exhibit spin crossover (SCO) behavior at around room temperature. Therefore, they are interesting for a variety of possible applications, and it is convenient to integrate them into polymers. Due to a reduction of the iron content and thus also ^57^Fe content in the sample through integration in polymers, Mössbauer measurements are only possible with greater difficulty or very long measurement times without expensive enrichment of the samples with ^57^Fe. So, other ways of improving the Mössbauer signal for these composite materials are necessary. Therefore, we pressed these composite materials to improve the Mössbauer spectra. In this study, we synthesized an iron(II) triazole spin crossover complex and an electrospun polymer complex composite nanofiber material including the same complex. For both products, Mössbauer measurements were performed at room temperature before and after using a press to show that the complex composite is not harmed through pressing. We investigate the influence of the pressing impact on the Mössbauer measurements in the context of measurement statistics and the measured signals. We show that pressing is not connected to any changes in the sample regarding the spin and oxidation state. We present that pressing improves the statistics of the Mössbauer measurements significantly. Furthermore, we use SEM measurements and PXRD to investigate whether or not the obtained fiber mats are destroyed in the pressing process.

## 1. Introduction

Regarding many research fields, Mössbauer spectroscopy has proven to be a reliable powerful tool for the analysis of samples [1,2]. ^57^Fe Mössbauer is used in many cases to analyze the spin and oxidation states of iron in samples [3,4,5]. Mössbauer spectroscopy can, for example, be used for the analysis of industrial samples and even for the analysis of future radioactive waste container materials [6]. It was also used during several Mars missions for the analysis of iron-rich soils and led to the mineralogical evidence of water on Mars [7,8,9].

Mössbauer spectroscopy is frequently used for the analysis of coordination compounds, like SCO compounds. This category of complexes exhibits a reversible spin state switching between high- and low-spin states through several different physical (e.g., temperature, light irradiation, pressure) or chemical stimuli (e.g., water, volatile organic compounds). In regards to application and, therefore, implementation, several possible matrices, like polymers and iron(II) triazole complexes, are often considered. These exhibit the SCO effect around room temperature and through other suitable stimuli [10]. Electrospun polymer fiber mats can be seen as a suitable example of matrices for these systems. Electrospinning is a versatile production technique to produce nanofibers out of polymer solutions. The nanofibers in this process exceed the sub-100 nm range but will still be called nanofibers in the aspect of engineering and the industry [3,11]. During the electrospinning process, an electrical potential (typically 5 and 30 kV) is applied between a grounded collector and a droplet of the solution in a syringe needle. As the Coulomb forces, due to the applied voltage acting on the droplet, overcome the surface tension of the solution, a fiber jet emerges from the Taylor Cone, and the conical meniscus is formed during the electrospinning process [12,13]. Due to the small size, the liquid fiber dries mid-air, and the remaining electrically charged nanofibers remain and discharge on the collector. In our case, we use a rotating drum collector to obtain aligned fibers, depending on the used velocity [3]. In the case of composite materials, like fiber mats, they are often difficult to obtain Mössbauer spectra are assignable to their containing iron species over a reasonable time frame without the substitution of the iron-containing material with ^57^Fe. The latter is known to be expensive regarding unavoidably larger samples for many conceivable composite materials. In recent studies, we realized that even longer measurement times do not significantly improve the detected signal for composite nanofibers with integrated SCO complexes. We realized that SCO behavior was at least maintained in these composites, but monitoring the spin state of the iron compound was difficult [14]. As the spin state and the oxidation are connected to the functionality of these composites, it is necessary to improve these kinds of measurements. Therefore, we decided to try to press the composite samples to have punctually more iron(II) of the complex in the beamline. We observed an improvement in the detected signal with a lower signal-to-noise ratio in comparison to the unpressed composite nanofiber material in a shorter amount of time. This could potentially lead to better measurement signals for other composite fiber materials in the future.

## 2. Materials and Methods

### 2.1. General

The iron(II)–triazole complexes have been synthesized with the following chemicals without further purification: Iron(II)chloride tetrahydrate (FeCl_2_ · 4H_2_O) (>99%) from Sigma-Aldrich (St. Louis, MO, USA); L-Ascorbic acid (>99%) from Carl Roth; 4-amino-1,2,4-triazole (99%) purchased from Thermo Scientific (Waltham, MA, USA); and Sodium 2-naphthalenesulfonate (98%) from Alfa Aesar (Haverhill, MA, USA).

The obtained composite fibers were electrospun using the following chemicals: polyacrylonitrile (PAN) 150,000 Mw from Sigma-Aldrich (St. Louis, MO, USA) and N,N-Dimethylformamide (DMF) from Carl Roth. 

The pressed samples were prepared using an electrohydraulic press from Mauthe with a force of 10^5^ N, as it is the maximum applicable force for this device.

### 2.2. Methods

#### 2.2.1. Synthesis of [Fe(Atrz)_3_](2ns)_2_

The necessary iron(II) salt iron(II)-2-naphthalenesulfonate hexahydrate (Fe(2ns)_2_ · 6H_2_O) for the complex synthesis was obtained through a previously performed synthesis [3]. The complex was then subsequently obtained, as formerly reported [14]. 

FeC_26_H_26_N_12_O_6_S_2_ · 4 H_2_O (molar mass 794.597 g mol^−1^): C, 38.90 (39.30); H, 4.07 (4.31); N, 21.13 (21.15) MIR (in cm^−1^): 474 (m), 502 (m), 552 (s), 560 (s), 568 (s), 622 (s), 647 (m), 675 (s), 748 (s), 768 (w), 819 (s), 865 (s), 906 (s), 944 (m), 956 (m), 981 (w), 1032 (s), 1063 (m), 1093 (s), 1138 (s), 1184 (s), 1271 (s), 1346 (w), 1383 (w), 1446 (m), 1504 (w), 1544 (w), 1593 (w), 1628 (m,broad), 3011 (w), 3060 (m), 3073 (w), 3134 (w), 3163 (m), 3214 (w), 3283 (m,broad), and 3498 (m,broad).

#### 2.2.2. Synthesis of Composite Fibers

**Figure 1:**
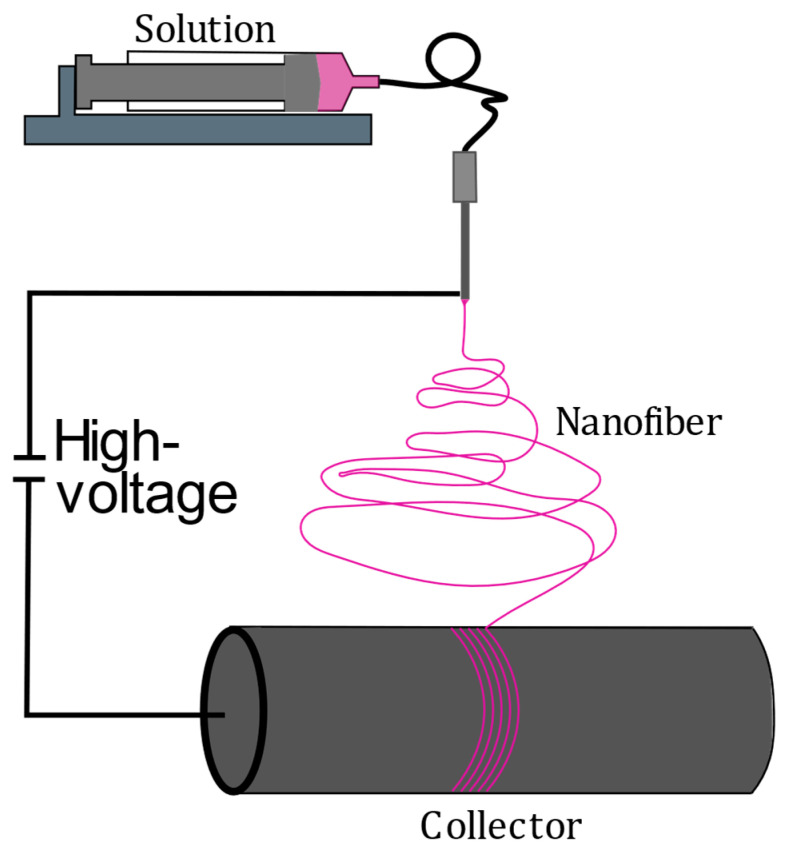
Schematic of the electrospinning process of the composite.

A solution was prepared by solving 0.395 g of [Fe(Atrz)_3_](2ns)_2_ with 0.254 g L-Ascorbic acid in 7 mL DMF, which was then sonicated for 1 h. In a separate solution, 1.012 g PAN was dissolved in 3 mL DMF and stirred overnight for 12 h to obtain a homogeneous solution. Next, both of the solutions were combined under heavy stirring. The solution was electrospun at 18 kV with a pumping rate of 0.55 mL h^−1^, a collector speed of 10 ms^−1^, a needle diameter of 0.8 mm, and a needle-to-collector distance of 20 cm at room temperature. The resulting fiber was collected on an aluminum foil applied on a rotating collector, as schematically shown in Figure 1.

### 2.3. Characterization

Infrared spectroscopy was performed to confirm the successful synthesis of the complex. Therefore, a Perkin-Elmer spectrum was used with the ATR method between 450 and 4000 cm^−1^.

CNH elemental analysis was performed using a Perkin-Elmer 2400 II CNH analyzer.

Mössbauer spectroscopy was performed at room temperature (19 °C) with a customized “WissEl” drive setup in transmission mode using a “MIMOS II” silicon pin detector [15,16]. Co-57 in a Rh matrix was used as the Mössbauer source. The chosen energy for the measurement was 14.4 keV. The calibration and fitting were performed with the program “Recoil”. All values are given in relation to α-Fe.

To measure the unpressed complex, 0.1 g of it was weighed and placed in our standard sample carrier (round with a 2 cm diameter). To measure the pressed composite, a tablet (1 cm diameter) was pressed at a force of 10^5^ N for 2:30 min. For each of the composites, 0.1 g was weighed. The unpressed composite was placed in a sample carrier (round with a 1 cm diameter). The morphology of the nanofibers and pressed samples were analyzed by Scanning Electron Microscopy (SEM) using a Carls Zeiss Supra 55VP.

For the measurement of the nanofiber diameter, Image J was used, and forty readings were performed from different SEM images.

Powder X-ray diffraction (PXRD) was used for the structural characterization of the complex, pristine, and pressed educts and the polymer, as well as the composite with pristine and pressed fibers using a Bruker D2 phaser. The samples were scanned from 10 to 90° 2θ with a stepping of 0.08° using a Cu-Kα (λ = 1.5406 Å) radiation.

## 3. Results

**Figure 2:**
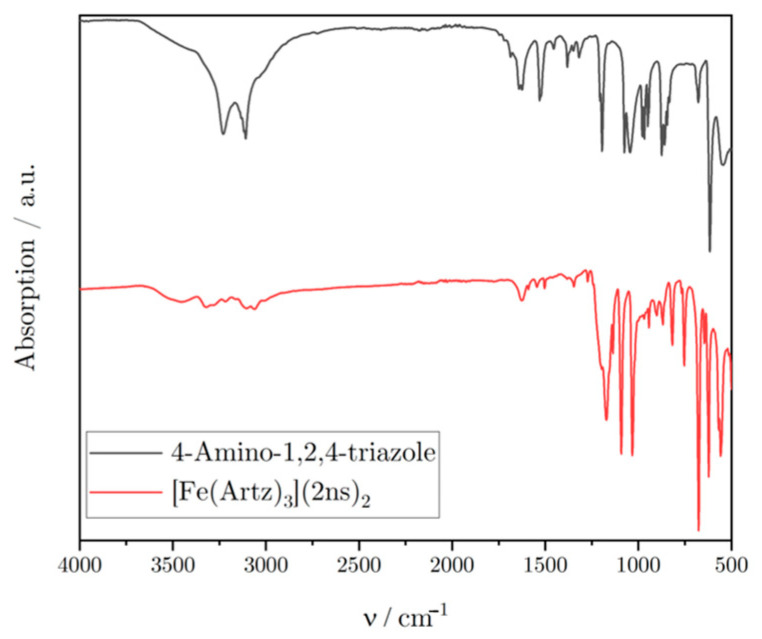
IR spectra of the ligand and the synthesized complex.

To analyze the obtained complexes and confirm a successful synthesis, IR spectroscopy, Mössbauer Spectroscopy, PXRD, and CHN elemental analyses were performed. The CHN elemental analysis proved that the fitting amounts of elements should have been in the sample with a certain amount of water molecules. The infrared spectrum further proved that the expected complex was synthesized. Bonds that are, for example, specific for the triazoles ring torsion are present at 622 cm^−1^, and other specific bonds are present for the 2ns anion, e.g., 1032 cm^−1^, which can be assigned to the sulfonate groups bonds. Figure 2 shows a comparison of the IR spectrum of the ligand 4-amino-1,2,4-triazole with the obtained complex. Similar bonds, for example, the bond at 622 cm^−1^, which can be assigned to the triazoles ring strain, are also visible in the spectrum of the pure ligand. Through this, it is visible that the obtained product is indeed the intended complex. Furthermore, the obtained complex had a characteristic pink color, which also indicated the successful synthesis. A reversible change in the color from pink to white was also possible through heating the sample, which is explained through the spin crossover phenomenon [17,18].

**Figure 3:**
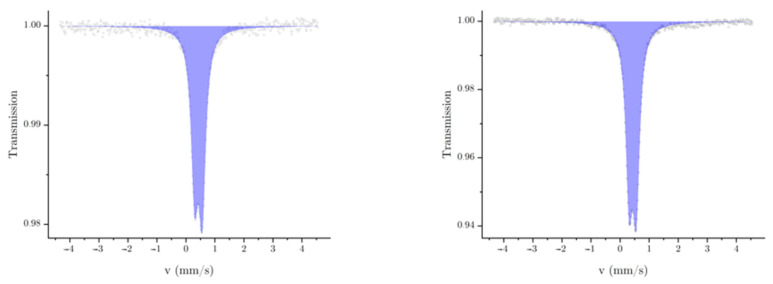
Mössbauer spectra of the pure unpressed (**left**) and pressed (**right**) complex.

In Figure 3, the Mössbauer spectra of the pure unpressed (left) and pressed (right) complex are shown. Both spectra show a single duplet signal, which can be assigned to iron(II) in the low-spin state, as the isomeric shift (δ) and the quadrupole splitting (ΔEq) are characteristic for iron(II) triazole complexes in this spin state [18]. The isomeric shift and the half width at half maximum (w) are similar in both spectra. The quadrupole splitting of the spectrum of the pressed complex is minimally smaller by 0.018 mm·s^−1^. The relatively small quadrupole splitting can be explained by a homogeneous ligand field around the iron centers. The spectrum of the unpressed material on its own speaks, therefore, to a successful synthesis of the complex. The asymmetry of the fits (a−/a+) is slightly different. A possible reason for this could be the different sample thicknesses after the pressing of the sample (all parameters of the fits are shown in Table 1). The asymmetries of the half width at half maximum (w−/w+) were fixed to 1. The statistic of the measurement of the pressed material is better with a visible improvement of the signal-to-noise ratio in roughly the same measurement time. The spectrum of the pressed material shows a decreased transmission and, therefore, a higher absorption, which can be explained by a higher amount of iron in the beamline. 

**Figure 4:**
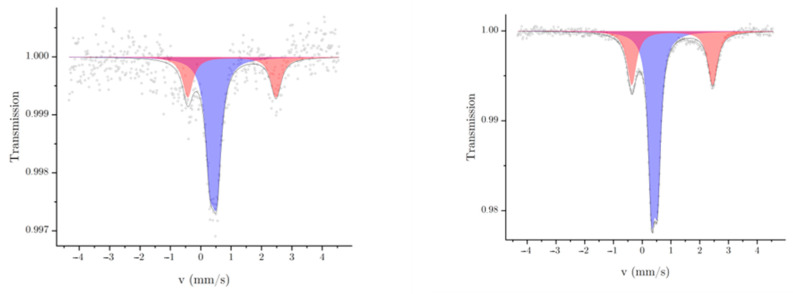
Mössbauer spectra of the unpressed (**left**) and pressed (**right**) composites.

To observe the spin state and oxidation state of the complex inside of the polymer before and after the pressing, Mössbauer spectroscopy was performed for both samples. Therefore, Figure 4 shows the Mössbauer spectra of the composites in their unpressed and pressed states. Both spectra show two duplet signals that can be assigned to iron(II) in the low-spin (LS) and high-spin (HS) states [18]. The LS state shows a small quadrupole splitting, which also can be explained in this case by the homogeneous ligand field in the complex inside the composite. In comparison to the unpressed material, a fraction of the HS state is visible in both spectra of the composite. The reason for this could be the interaction between the polymer and the complex [13]. The HS state has a different quadrupole splitting than the LS, which is due to the more inhomogeneous ligand field. The lower statistics of the Mössbauer spectrum of the unpressed sample can be clearly seen, even though the measurement time of the unpressed composite was significantly longer than the measurement time of the pressed sample. In this context, it can be emphasized that the measurement of the unpressed composite was nearly twice as long. The area percentages are comparable in both spectra, but the error of the fit is relatively high at ±11% for the unpressed composite due to the poorer statistics. The poorer statistics also lead to higher deviations of the fits for the other parameters. Within the limits of the deviations, however, the values are all comparable. The isomeric shift (δ) of the low-spin and high-spin components of the measurements are matching. The quadrupole splitting (ΔEq) and the other parameters are also similar as well (Table 2). As also seen in the case of the pure pressed and unpressed complex, the transmission in the spectrum of the pressed composite was decreased and, subsequently, the absorption increased. This is also explainable by more iron(II) in the beamline. The errors of the parameters of the Mössbauer fits of the unpressed composite are significantly greater than those of the pressed composite, which can be explained by the lower statistics. 

**Figure 5:**
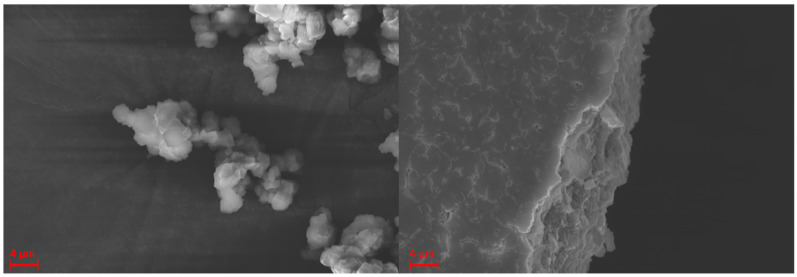
SEM Imaging of the [Fe(Atrz)_3_](2ns)_2_ powder and [Fe(Atrz)_3_](2ns)_2_ pressed pellet at 15 kV acceleration voltage.

Additionally, for the Mössbauer measurements, SEM images of the complex and the composite fiber material were recorded. Figure 5 shows SEM images of the [Fe(Atrz)_3_](2ns)_2_ complex as powder and as a pressed sample. As a powder, it is visible that the complex comes as grains with an average particle size of 1.2 µm, with some grains reaching up to 4 µm. This can be explained by the agglomeration of particles. In contrast, such small grains are not reliably measurable for the pressed sample. The surface of the pellet is rough with many fissures and uneven edges, and single particles are not visible.

**Figure 6:**
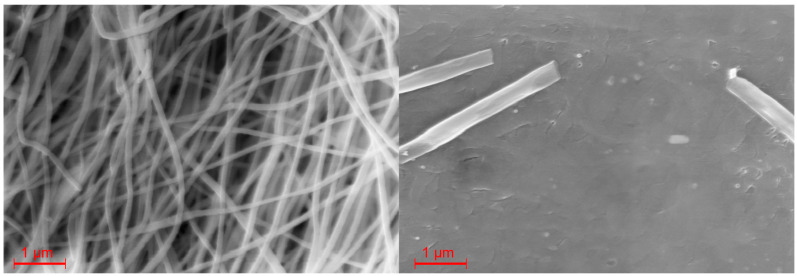
SEM images of spun PAN [Fe(**A**trz)_3_](2ns)_2_ nanofibers and pressed nanofiber surface at 15 kV acceleration voltage.

Figure 6 depicts the SEM images of the composite in the pressed and unpressed state. The nanofibers have an average diameter of 345 nm. In the overview image, some minor beadings are visible. Those are present due to some inconsistencies during the electrospinning process and can consist of an elevated amount of both PAN and [Fe(Atrz)_3_](2ns)_2_. In comparison, the SEM picture of a pressed composite pellet shows that only a few nanofibers are visible on the surface, and some of them seem wider. This can be explained by the flat squashing of the fibers during the pellet preparation process. Overall, the pellet of the composite is smoother than the pellet of the pure complex, which is due to the polymer’s properties. 

**Figure 7:**
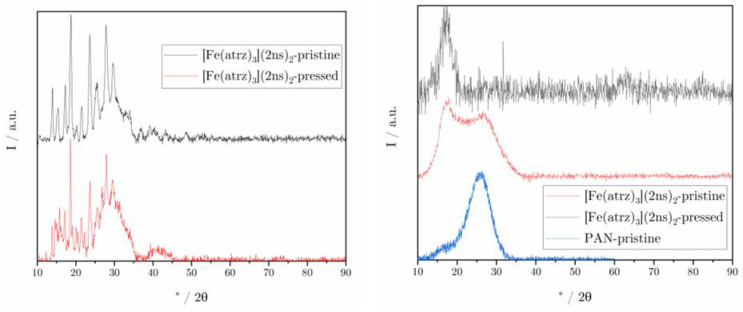
PXRD pattern of pristine and unpressed complex (**left**) and the pattern of pristine and pressed composite and pristine PAN for comparison (**right**).

For further structural analysis, PXRD was performed for the complex and the composite in the pressed and unpressed state. The measured diffraction patterns of these are shown in Figure 7. The refraction patterns of the complex show only small changes after pressing, and the most relevant reflexes are still present. Therefore, no significant change can be observed regarding the crystal structure of the complex, even though some of the reflexes are less visible after pressing. The differences in these patterns can be explained by a preferential direction in the crystal structure. The refraction pattern of the unpressed composite shows a broad reflex, which is the major visible reflex in the pattern. The pressed composite has a second broad reflex, which can be assigned to the PAN as it is a major part of the composite and has a characteristic reflex at that position. This is also supported by the direct comparison with the pure PAN. The PAN reflex is presumably more visible after pressing due to an increased crystallinity. The pressed and unpressed composite generally show a more amorphous pattern in comparison to the pressed and unpressed complex. This is also due to the semi-crystalline properties of the PAN.

## 4. Discussion

The Mössbauer spectra and the resulting Mössbauer parameters show no significant change between the pressed and unpressed pure complex. This indicates that the environment of the iron nuclei has not changed significantly. So, the complex is not destroyed by notably pressing or changing its spin state. Only an increase in the absorption can be observed in the case of the pressed material, which is explainable by potentially more iron(II) in the beamline. In the case of the composite, the differences between the Mössbauer fits are intensified due to the poorer statistics of the measurement of the unpressed sample, but taking into account the larger errors, the values match. So, the pressing of the composites, therefore, has no significant influence on the Mössbauer parameters of the measured composite and can, therefore, be used as a sample preparation to obtain a significant improvement in the statistics for Mössbauer measurements while simultaneously reducing the measuring time. The transmission was also reduced and, therefore, the absorption increased in the case of the pressed composite due to more iron(II) in the beamline. The unpressed composite had a measurement time of roughly 11 days and the pressed composite was measured for roughly 6 days. Looking at the statistics of the measurement of the unpressed sample, it would have been necessary to at least measure the sample for double the amount of time (22 days), suggesting that the pressing of the composite accelerated the measurement by at least three times. In the case of our composite, the time of a normal unpressed sample measurement would take as long as nearly one-tenth of the half-life time of our used Mössbauer source. 

The SEM images show a clear difference between pristine and pressed complex particles and nanofiber composite. The pressed pure complex can be ground back into smaller grains, but the composite cannot be used anymore for further fiber-related measurements, as the nanofiber structure is irreversibly destroyed. The complex in the fiber itself though is, according to Mössbauer data, not affected by the pressing, as mentioned before. The PXRD patterns of the complex showed no significant difference, which was also confirmed by the Mössbauer spectra. The minor changes in the patterns are, therefore, not linked to oxidation as the Mössbauer spectra, and both cases only showed the presence of iron(II). The XRD pattern of the composite shows a more amorphous structure, as only the main reflex from the complex remains. The additional PAN reflex is only visible in the pressed composite, which could be due to the semi-crystalline properties of the polymer and the small sample thickness for the measurements. The broadening of the main complex reflex is caused by the smaller crystallites along the nanofiber structure. 

For similar complex systems, pressing is, therefore, a good way of preparing samples for Mössbauer measurements for better statistics. For other complex systems than iron(II) triazole complexes, it would have to be tested whether or not pressing would have a significant effect on the spin state and the resulting Mössbauer parameters and destroy the complex. 

## 5. Conclusions

To study the principal behavior and changing of the spin states of a triazole complex in a spun polymer fiber, Mössbauer spectroscopy is a powerful method. Due to the low iron content, the statistic of the unpressed composite is poor and the measurement time is long. An easy way to improve the quality of the Mössbauer spectra by pressing the samples was shown. Both the pure triazole complexes and the triazole complexes in a spun polymer fiber are not significantly affected by pressing. This increases the absorption due to a higher amount of iron in the beamline. However, it has to be mentioned that the SEM pictures show that the composite pellet cannot be utilized after pressing. For similar composites, pressing is, therefore, a good way of preparing samples for Mössbauer measurements. For other systems, it would have to be tested whether pressing would have a significant effect on the spin state and the oxidation state of the complex in the composite system. The PXRD patterns showed that no major structural changes have occurred by pressing the powder and composite samples. The minor visible changes cannot be assigned to oxidation processes, as only iron(II) was observed in the Mössbauer spectra. Therefore, they can potentially be assigned to preferential directions in crystalline or semi-crystalline structures. We generally aimed to improve Mössbauer signals in composite fibers and showed that it is possible by simply pressing the fiber mats instead of relying on exaggerated unreasonable measuring sessions or expensive enrichments with ^57^Fe.

## Figures and Tables

**Table 1:** Mössbauer fit parameters of the spectra of the pure complex, unpressed (1) and pressed (2). Fixed values are indicated with *.

Sample	δ/mm·s^−1^	ΔEq/mm·s^−1^	w/mm·s^−1^	a−/a+	w−/w+	Area/%	t/s
1	0.431 ± 0.004	0.259 ± 0.005	0.145 ± 0.004	0.882 ± 0.045	1 *	100	175,261
2	0.434 ± 0.002	0.241 ± 0.002	0.140 ± 0.002	0.944 ± 0.025	1 *	100	166,234

**Table 2:** Mössbauer fit parameters of the spectra of the unpressed (1) and pressed (2) composites. Fixed values are indicated with *.

Sample		δ/mm·s^−1^	ΔEq/mm·s^−1^	w/mm·s^−1^	a−/a+	w−/w+	Area/%	t/s
1	LS	0.419 ± 0.044	0.222 ± 0.048	0.180 ± 0.039	0.861 ± 0.548	1 *	69.4 ± 7.3	956,825
	HS	1.025 ± 0.052	2.905 ± 0.105	0.206 ± 0.087	1 *	1 *	31 ± 11	
2	LS	0.429 ± 0.004	0.205 ± 0.004	0.140 ± 0.004	1.108 ± 0.065	1 *	65.7 ± 0.9	526,180
	HS	1.045 ± 0.006	2.816 ± 0.013	0.196 ± 0.010	1 *	1 *	34.3 ± 1.5	

## Data Availability

The data and materials are available from the corresponding author upon reasonable request.

## References

[1] Gütlich P. (2012). Fifty years of Mössbauer spectroscopy in solid state research—Remarkable achievements, future perspectives. Z. Anorg. Und Allg. Chem..

[2] Gütlich P., Bill E., Trautwein A.X. (2011). Mössbauer Spectroscopy and Transition Metal Chemistry.

[3] Kilic M.S., Brehme J., Pawlak J., Tran K., Bauer F.W., Shiga T., Suzuki T., Nihei M., Sindelar R.F., Renz F. (2023). Incorporation and deposition of spin crossover materials into and onto electrospun nanofibers. Polymers.

[4] Renz F., Spiering H., Goodwin H., Gütlich P. (2000). Light-perturbed hysteresis in an iron(II) spin-crossover compound observed by the Mössbauer effect. Hyperfine Interact..

[5] Heyer L., Dreyer B., Preiss A., Menze M., Klimke S., Jahns M., Sindelar R., Klingelhöfer G., Costa B.F.O., Renz F. (2016). Mössbauer investigation of novel pentadentate schiff base complexes. Hyperfine Interact..

[6] Kaufhold S., Klimke S., Schloemer S., Alpermann T., Renz F., Dohrmann R. (2020). About the Corrosion Mechanism of Metal Iron in Contact with Bentonite. ACS Earth Space Chem..

[7] Klingelhöfer G., Morris R.V., Bernhardt B., Rodionov D.S., de Souza P.A., Squyres S.W., Foh J., Kankeleit E., Bonnes U., Gellert R. (2003). Athena MIMOS II Mössbauer spectrometer investigation. J. Geophys. Res. Planets.

[8] Klingelhöfer G., Fegley B., Morris R.V., Kankeleit E., Held P., Evlanov E., Priloutskii O. (1996). Mineralogical analysis of Martian soil and rock by a miniaturized backscattering Mössbauer spectrometer. Planet. Space Sci..

[9] Klingelhöfer G., Morris R.V., Bernhardt B., Schröder C., Rodionov D.S., de Souza P.A., Yen A., Gellert R., Evlanov E.N., Zubkov B. (2004). Jarosite and Hematite at Meridiani Planum from Opportunity’s Mössbauer Spectrometer. Science.

[10] Sugahara A., Kamebuchi H., Okazawa A., Enomoto M., Kojima N. (2017). Control of Spin-Crossover Phenomena in One-Dimensional Triazole-Coordinated Iron(II) Complexes by Means of Functional Counter Ions. Inorganics.

[11] Kilpakjan S., Schmid S.R. (2019). Manufacturing Engineering and Technology.

[12] Baumgarten P.K. (1971). Electrostatic spinning of acrylic microfibers. J. Colloid Interface Sci..

[13] Taylor G.I. (1969). Electrically driven jets. Proc. R. Soc. London. Ser. A Math. Phys. Sci..

[14] Brehme J., Kilic M.S., Pawlak J., Renz F., Sindelar R.F. (2024). Soluble molecular switches in electrospun nanofibers. Hyperfine Interact..

[15] Jahns M., Klimke S., Natke D., Sindelar R., Schrewe U., Patzke R., Renz F. (2019). An Arduino based Mössbauer spectrometer. Nucl. Instrum. Methods Phys. Res. Sect. A.

[16] Pawlak J., Beyki M., Jahns M., Bauer F., Sindelar R., Patzke R., Renz F. (2023). 2D data processing with MIMOS II. Hyperfine Interact..

[17] Lavrenova L.G., Shakirova O.G., Ikorskii V.N., Varnek V.A., Sheludyakova L.A., Larionov S.V. (2003). ^1^A_1_ ⇄ ^5^T_2_ Spin Transition in New Thermochromic Iron(II) Complexes with 1,2,4-Triazole and 4-Amino-1,2,4-Triazole. Russ. J. Coord. Chem..

[18] van Koningsbruggen P.J., Garcia Y., Codjovi E., Lapouyade R., Kahn O., Fournès L., Rabardel L. (1997). Non-classical FeII spin-crossover behaviour in polymeric iron(II) compounds of formula [Fe(NH2trz)3]X2·xH2O (NH2trz = 4-amino-1,2,4-triazole; X = derivatives of naphthalene sulfonate). J. Mater. Chem..

